# Essential Oil Yield, Composition, and Bioactivity of Sagebrush Species in the Bighorn Mountains

**DOI:** 10.3390/plants11091228

**Published:** 2022-05-01

**Authors:** Valtcho D. Zheljazkov, Charles L. Cantrell, Ekaterina A. Jeliazkova, Tess Astatkie, Vicki Schlegel

**Affiliations:** 1 Crop and Soil Science Department, Oregon State University, 3050 SW Campus Way, Corvallis, OR 97331, USA; ekaterina.jeliazkova@oregonstate.edu; 2 Natural Products Utilization Research, USDA-Agricultural Research Service, University of Mississippi, University, MS 38677, USA; charles.cantrell@usda.gov; 3 Faculty of Agriculture, Dalhousie University, Truro, NS B2N 5E3, Canada; astatkie@dal.ca; 4 Department of Food Science and Technology, University of Nebraska-Lincoln, 326 Food Technology Complex, Lincoln, NE 68583, USA; vschlegel3@unl.edu

**Keywords:** *Artemisia tridentata*, *A. longifolia*, *A. ludoviciana*, *A. cana*, camphor, antioxidant, eucalyptol, cis-thujone, α-pinene, α-necrodol-acetate

## Abstract

Sagebrush (*Artemisia* spp.) are dominant wild plants in large areas of the U.S., Canada and Mexico, and they include several species and subspecies. The aim was to determine if there are significant differences in essential oil (EO) yield, composition, and biological activity of sagebrush within the Bighorn Mountains, U.S. The EO yield in fresh herbage varied from 0.15 to 1.69% for all species, including 0.25–1.69% in *A. tridentata* var. *vaseyana*, 0.64–1.44% in *A. tridentata* var. *tridentata*, 1% in *A. tridentata* var. *wyomingensis*, 0.8–1.2% in *A. longifolia*, 0.8–1% in *A. cana*, and 0.16% in *A. ludoviciana*. There was significant variability in the EO profile between species, and subspecies. Some EO constituents, such as α-pinene (0–35.5%), camphene (0–21.5%), eucalyptol (0–30.8%), and camphor (0–45.5%), were found in most species and varied with species and subspecies. The antioxidant capacity of the EOs varied between the species and subspecies. None of the sagebrush EOs had significant antimicrobial, antimalarial, antileishmanial activity, or contained podophyllotoxin. Some accessions yielded EO with significant concentrations of compounds including camphor, eucalyptol, cis-thujone, α-pinene, α-necrodol-acetate, fragranol, grandisol, para-cymene, and arthole. Therefore, chemotypes can be selected and possibly introduced into culture and be grown for commercial production of these compounds to meet specific industry needs.

## 1. Introduction

The genus *Artemisia*, distributed mostly in the Northern hemisphere, comprises small herbs and shrubs of over 500 species occurring in North America, Europe, and Asia [1]. Sagebrush (*Artemisia* spp.) are woody shrub species of the genus and the dominant wild species in large areas of Western United States, Canada, and Mexico. They occupy around 69 million ha in the Western United States alone [2,3]. The woody sagebrush include several species such as big sagebrush (*Artemisia tridentata* Nutt.), black sagebrush (*Artemisia nova* A. Nelson), silver sagebrush (*Artemisia cana* Pursh), several subspecies and hybrids (crosses) between these [4], distributed in various states and provinces in North America [5]. Sagebrush communities contribute to the survival of various wildlife species, especially the greater sage-grouse (*Centrocercus urophasianus*) and the Gunnison sage-grouse (*Centrocercus minimus*) [3,6,7,8,9]. Pronghorn antelope (*Antilocapra americana*) is thought to have evolved with sagebrush and is now perhaps the only animal species to browse it extensively, whereas sage-grouse is endemic for sagebrush ranges. Other bigger animals, such as mule deer (*Odocoileus hemionus*), elk (*Cervus canadensis*), and bighorn sheep (*Ovis canadensis*), browse sagebrush. It may also be browsed by domestic sheep and cattle especially during the winter months, when the ground is covered with snow and there is limited other forage available [10,11]. The grazing and browsing of big and small sagebrush by wild ungulates and cattle have been shown to modify density of sagebrush species and to shift the species composition [12]. Sagebrush density habitat changes have been shown to affect mortality of pronghorn [13]. The importance of sagebrush rangelands for many animal and bird species has been outlined in the literature; sagebrush affords habitats for obligate species (the ones that live only in sagebrush ecosystems) and facultative species (the animals that may use sagebrush ecosystems and other ecosystems) [3]. 

In addition to providing cover for wildlife, sagebrush is extensively used in the reclamation of disturbed lands, especially in oil and natural gas exploration and development areas, which started in the 1920s and peaked in 2010–2013. Oil and gas exploration, renewable energy such as wind and solar developments, and intensification of recreational activities have all been affecting sagebrush and associated species, such as sage-grouse, habitat [14,15]. Fragmentation of habitat by anthropogenic activities such as mining and energy developments has generally had a negative effect on the sagebrush and sage-grouse population in Wyoming, where 37% of sage-grouse population resides [14,15].

Sagebrush species have been used extensively by native peoples in North America as medicinal plants [16]. A comprehensive review on sagebrush species research conducted in North America provided insights into the evolution, botany and taxonomy, phytochemical complexity, and also pharmacological findings [17]. Sagebrush EO and extracts have shown antifungal activity [16,18,19]. A study with extracts from 100 medicinally active plants reported that extracts of aerial parts of *A. ludoviciana* and *A. tridentata* (among other species) had significant fungal inhibitory activity [16]. 

Sagebrush plant species contain substantial amounts of EO with a strong specific aroma; however, the EO is toxic [20]. The EO of aboveground plant parts of sagebrush species, including *A. tridentata*, has been investigated in the past [21,22]. However, the EO yield, composition, and antimicrobial activity of different sagebrush species are yet to be fully characterized. The hypothesis of this study was that there would be significant between-species (interspecies) and within-species (intraspecies) variations of EO yield, composition, and antimicrobial activity. The objective of this study was to assess sagebrush species in the Bighorn Mountains area of Wyoming, and their EO yield, composition, and bioactivity.

## 2. Results and Discussion

There were two separate collections of sagebrush (in 2011 and in 2014) that were treated as two different studies. Response variables were compared within each collection.

### 2.1. The 2011 Collections

In the 2011 collection, 13 accessions were identified as *A. tridentata* Natt. var. *vaseyana*, and were collected from elevations ranging from 1907 to 2980 m; 3 accessions were *A. longifolia*, collected at elevations ranging from 1147 to 1166 m; 2 accessions were *A. cana* var. *cana*, collected at elevations of 1292 and 1333 m; another 2 accessions were *A. tridentata* var. *tridentata*, collected at 2141 and 2299 m; 1 accession was identified as *A. ludoviciana* ssp. *ludoviciana*, collected at 1643 m; 1 accession identified as *A. tridentata* var. *wyomingensis*, collected at 1453 m (Table 1).

#### 2.1.1. *Artemisia tridentata* var. *vaseyana*

The chemical constituents found in the oil of the *A. tridentata* var. *vaseyana* accessions ranged from 13 to 48 per accession (Table S1). However, the amassed number of identified constituents amounted to 116 different constituents, indicating significant variability in the EO profile within the *A. tridentata* var. *vaseyana* subspecies. The major constituents present in the EO of *A. tridentata* var. *vaseyana* were α-pinene at 35.5% in accession #210, camphor at 45.4% for accession #212, eucalyptol at 17.9% for accession #211, chrysanthenone at 17.9% for accession #201, fragranol at 20.3% and grandisol at 36.2% for accession #203 (Table S1). A previous study found 42 constituents in the EO obtained by steam distillation from leaves and branches of *A. tridentata*, with camphor, camphene, and 1,8-cineole (eucalyptol) accounting to 28.6, 16.9, and 13.2% of the total oil [23].

#### 2.1.2. *Artemisia tridentata* var. *tridentata*

The chemical constituents found in the EO of *A. tridentata* var. *tridentata* are listed in Table S2 and were 24 and 27 for accession #221 and #222, respectively. Camphor, eucalyptol, chrysanthenone, and camphene were the major EO constituents for accession #221. For accession #222, the major EO constituents were camphor, camphene, eucalyptol, and α-pinene (Table S2).

#### 2.1.3. *Artemisia tridentata* var. *wyomingensis*

The 18 EO constituents found in the oil of *A. tridentata* var. *wyomingensis* accession are listed in Table S3. Camphor, arthole, α-santoline alcohol, and eucalyptol were the major constituents at 20.6, 17.4, 14.8, and 13.0%, respectively.

#### 2.1.4. *Artemisia cana*

Among the 30 identified constituents in the EO of *A. cana*, camphor, eucalyptol, α-santoline alcohol, and arthole were the major constituents in accession #219 at 35.3%, 15.3%, 11.3%, and 9.0%, respectively (Table S4). The major constituents in the EO of accession #220 were camphor, eucalyptol, camphene, and arthole at 40.6%, 20.5%, 6.6%, and 6.2% of the total oil, respectively. 

#### 2.1.5. *Artemisia longifolia*

The chemical constituents found in the EO of *A. longifolia* are listed in Table S5. Among the 30 identified constituents, 11, 12, and 9 constituents were present above 1% in the oil of accessions #216, 217, and 218 accounting for 90.9%, 90.9%, and 91.4%, respectively. Eucalyptol at 30.8% was the major constituent in the EO of accession #216, while camphor was the major constituent at 27.7% and 43.4% in the EO of accessions #217 and #218, respectively.

#### 2.1.6. *Artemisia ludoviciana* ssp. *ludoviciana*

Among the 33 constituents found in the EO of *A. ludoviciana* ssp. *ludoviciana* accession, 9 were present at concentrations of above 1% and accounted for 90% of the oil (Table S6). Among these 9 constituents, camphor, eucalyptol, camphene, and borneol were present at concentrations of 46.2%, 17.9%, 13.3%, and 4.6%, respectively.

### 2.2. The 2014 Collections

In the 2014 collection, 3 accessions were identified as *A. tridentata* var. *vaseyana*, collected at elevations of 2172, 2300, and 2895 m; 2 accessions were *A. tridentata* var. *wyomingensis*, collected at elevations of 1192 and 2524 m; and 7 accessions as *A. cana* var. *cana*, collected at elevations ranging from 1105 to 1136 m (Table 1).

#### 2.2.1. *Artemisia tridentata* var. *vaseyana*

The number of identified chemical constituents found in the EO of *A. tridentata* var. *vaseyana* leaves obtained by hydro-distillation were 19, 28, and 9 for accessions #259, 261, and 262, respectively (Table S7). Artemiseole at 42.8%, camphor at 53.6%, and cyclooctadiene at 38.3% were the major constituents in the oils of accessions #259, 261, and 262, respectively.

#### 2.2.2. *Artemisia tridentata* var. *wyomingensis*

There were 27 identified constituents in the EO obtained by hydro-distillation of *A. tridentata* var. *wyomingensis* inflorescences. However, the identified constituents in the EO obtained by hydro-distillation of leaves were 13 for accession #251 and 27 for #260, with 7 out of these 13 constituents and 14 out of the 27 constituents unique to each accession (Table S8). Artemiseole at 32.6% and 26.3% was the major constituent in the EO of the leaves or inflorescences, respectively, for accession #251. Cis-thujone at 71% was the major constituent in the oil obtained from the leaves of accession #260.

#### 2.2.3. *Artemisia cana* var. *cana*

The 28 identified constituents in the EO of either leaves or inflorescences of *A. cana* var. *cana* accessions obtained by hydro-distillation are presented in Table S9. Camphor and eucalyptol were the major EO constituents for both leaves and inflorescences of *A. cana* Pursh var. *cana* accessions, except for accession #253. The major oil constituents for the leaves and inflorescences of accession #253 were ortho-cymene/para-cymene and α-phellandrene. Eucalyptol and camphor were the two major EO constituents in *A. cana* flowers, leaves, and stalks, collected from the Central Alberta Prairies, western Canada, although in different concentrations [24]. 

Overall, the results from this study demonstrated significant variation in EO content and composition in sagebrush species and subspecies collected from the Bighorn Mountains in Wyoming. Previous research from Oregon reported differences in EO composition between the three main subspecies of *A. tridentata*: *wyomingensis*, *tridentata*, and *vaseyna* [21]. The major constituents were methacrolein, camphene, artemiseole, eugenol, and artemisia ketone, with wide variations between and within the subspecies [21]. The wide variation in the concentration of individual chemical constituents between and within a subspecies is generally consistent with the results from this study. However, the chemical profile of the three *Artemisia tridentata* subspecies presented in the previous research from Oregon [21] is different from the ones in this study, which underlines the possibility for the existence of even greater chemical diversity among *A. tridentata* subspecies. 

Earlier report on *A. tridentata* identified camphor (40–45%) and eucalyptol (1,8-Cineole), α-pinene, β-pinene, camphene, thujone, and α-phellandrene being the major constituents; however, the exact subspecies was not identified [25].

The EO of *A. ludoviciana* var. *latiloba* from South Dakota was characterized with high concentration of oxygenated monoterpenes, such as camphor (20%), borneol, (around 15%), and eugenol (around 10%); however, it was obtained from a single accession [26]. Eugenol and camphor were also among the major constituents of the EO of *A. longifolia* and *A. ludoviciana*, and the latter contained significant amount of davanone [19].

### 2.3. Essential Oil Yield

**Figure 1:**
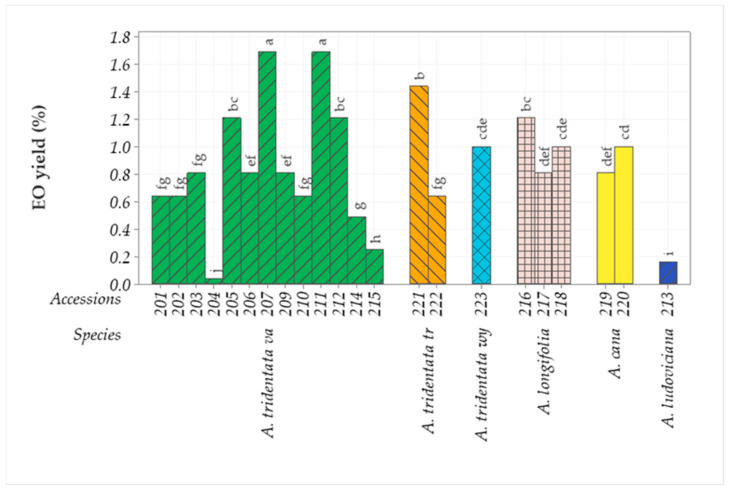
Mean EO yield (%) from six species and their accessions collected in the fall of 2011 from the Bighorn Mountains in Wyoming. Means sharing the same letter are not significantly different at the 5% level. *A. tridentata va* = *A. tridentata* Nutt. var. *vaseyana*; *A. tridentata tr* = *A. tridentata* Nutt. var. *tridentata*; *A. tridentata wy* = *A. tridentata* Nutt. var. *wyomingensis*.

The EO yield of the species and subspecies collected in 2011 varied widely, from 0.15% to 1.69% in fresh herbage within *Artemisia* spp. (Figure 1). Within *A. tridentata* var. *vaseyana* alone, the EO yield of fresh herbage varied from 0.25 to 1.69%. The EO yield within *A. longifolia* varied from 0.8 to 1.2%, whereas the EO yield in *A. cana* was 0.8–1% (not significantly different within the species), while the EO yield of *A. ludoviciana* was only 0.16%, in *A. tridentata* var. *tridentata* EO the yield was 0.64–1.44% and was 1% in *A. tridentata* var. *wyomingensis* (Figure 1). The EO yield of the species and subspecies collected in 2014 varied within and between species (Table S10). EO obtained by hydrodistillation of *A. tridentata* var. *vaseyana* leaves was 0.23% on average and varied from 0.06 to 0.42% (Table S10). Essential oil obtained by hydrodistillation of *A. tridentata* var. *wyomingensis* leaves or inflorescences was 0.89 and 1.15%, respectively (Table S10). The EO obtained from *A. cana* var. *cana* leaves was 0.47% on average and varied from 0.04 to 0.96%, while the average EO content of inflorescences was 0.61% and varied from 0.17 to 1.10% (Table S10). 

### 2.4. The Essential Oil Constituents Found in All Artemisia Species

Alpha-pinene was found in the EO of most species and subspecies with the exception of two accessions of *A. tridentata* var. *vaseyana*, #205 and 206, collected at elevations of 2980 and 2825 m, respectively. The concentration of α-pinene in the oil varied from undetectable amounts to 35.5% in *A. tridentata* var. *vaseyana*; the concentration of this monoterpene in the oils of the other Artemisia species did not vary significantly within a species (Table 2). Alpha-pinene, an alkene (C_10_H_16_), is a major constituent in orange peel EO, but also in the EO of many other species ranging from *Cannabis sativa*, *Rosemarinus officinalis* to *Pinus* ssp. [27,28,29]. Mercier et al. (2009) [28] have reviewed the many biological effects of α- and β-pinenes (in turpentine and its fractions). Table 3 shows the square root of Mean Squares Error (MSE) of the studied variables that estimates the common standard deviation (σ).

Similar to α-pinene, camphene (another monoterpene) was found in all *Artemisia* species and subspecies with the exception of the same two accessions of *A. tridentata* var. *vaseyana* (#205 and 206). Camphene concentrations varied from undetectable amounts to 13.8% within *A. tridentata* var. *vaseyana*, from 10.5 to 21.5% in *A. tridentata* var. *tridentata*, and was 6.6% in *A. tridentata* var. *wyimingensis*. Camphene concentration was 5.8–7% in *A. longifolia*, 6.6–7.4% in *A. cana*, and 13.3% in *A. ludoviciana*, and did not vary significantly within these species. 

Similar to α-pinene and camphene, eucalyptol was found in all *Artemisia* species and subspecies with the exception of the same two accessions #205 and 206 of *A. tridentata*. The concentration of eucalyptol varied from undetectable amounts to 30.8%; its concentration in *A. tridentata* was 0–21.2%, 24.6–30.8% in *A. longifolia*, 15.3–20.5% in *A. cana*, and 17.9% in *A. ludoviciana* (Table 2). The monoterpenoid eucalyptol (1,8 cineole), is a common EO constituent in other plant species and especially in eucalyptus (*Eucalyptus globulus*), *Artemisia vestita*, bay laurel (*Laurus nobilis*), but also in a number of other plants such as ginger (*Zingiber officinale*) and even a chemotype of lavender (*Lavandula stoechas*) [27,30,31,32,33,34]. Eucalyptol has wide applications as flavoring agent in consumer products such as mouthwashes, in perfumery and cosmetics, in cigarettes, and also in food products at low concentrations. Research has shown that eucalyptol has anti-inflammatory and anti-depressive effects [27].

Similar to α-pinene, camphene, and eucalyptol, camphor was found in all *Artemisia* species and subspecies with the exception of the same two accessions #205 and #206 of *A. tridentata*. Overall, the concentration of camphor varied from undetectable amounts to 45.5% in the oil of *A. tridentata* var. *vasseyana*, from 24.2 to 43.4% in the oil of *A. longifolia*, 35.2–40.6% in the oil of *A. cana* (Table 2). The waxy aromatic solid, camphor, a terpenoid (C_10_H_16_O), is isolated from wood of camphor tree, *Cinnamomum camphora*, and is also found in EO from other species such as *Cedrus libani*, *Ocimum kilimandscharicum*, *A. annua*, *A. vestita*, *Piper angustifolium*, *Sassafras albidum*, and *Rosmarinus officinalis* among others. Camphor has been known for centuries as an aromatic substance, and it has been used in ancient China and Japan, in other Asian countries and in Europe medicinally, but also in culinary applications and cosmetics [35]. It is a toxic substance with various biological activities ranging from antimicrobial, antiviral, insecticidal, and antitussive to anticancer activities [35,36]. 

Trans-α-necrodol-acetate, fragranol, and grandisol were found in the EOs of the collected accessions #201, 202, 203, 205, and 206 of *A. tridentata* var. *vasseyana* (Table 4). Additionally, trans-α-necrodol-acetate was found in the collected accessions #214 and 215 of *A. tridentata* var. *vasseyana.* The concentration of trans-α-necrodol-acetate varied from undetectable amounts (in most accessions) to 45.1% in *A. tridentata* var. *vasseyana;* this compound was not found in the oils of the other species (Table 4). Alpha-necrodol acetate (C_12_H_20_O_2_) is a rather rare constituent in plants and has been reported in the EO of two other plant species, *Lavandula suisieri* [37,38] and *Evolvulus alsinoides* (slender dwarf morning glory) [39].

The concentration of fragranol varied from 5.7 to 20.3% in the above-mentioned collected accessions of *A. tridentata* var. *vasseyana*, whereas the concentration of grandisol varied from 6.5 to 36.2% in the same accessions (Table 4). The monoterpene grandisol (C_10_H_18_O) is used as pheromone (sex attractant) for agricultural pests such as cotton boll weevil (*Anthonomus grandis*) and other weevils [40]. It has been reported in the EO of other *Artemisia* species such as *A. vestita* [32] and also in *Achillea falcata* [41]. 

Trans-arbusculone was found in the oils of only two collected accessions (#209 and 210) of *A. tridentata* var. *vasseyana*, and its concentration was 2.3–3.1% of the oil. Trans-pinocarveol was found in four accessions (#207, 209, 210, and 215) of *A. tridentata* var. *vasseyana* and its concentration varied from 2 to 24.8% of the oil. 

Chrysanthenone was found in the oils of three accessions (#201, 202, and 207) of *A. tridentata* var. *vasseyana*, and its concentration ranged from 1.2 to 21.6% of the oil (Table 4).

Borneol was not found in *A. tridentata*, it was only present in the oils of *A. longifolia*, *A. cana*, and *A. ludoviciana*, and its concentration was 2.3–2.6% in the oils of *A. longifolia* and *A. cana* and 4.6% in the oil of *A. ludoviciana* (Table 5). Pinocarvone was found in six accessions of *A. tridentata* var. *vasseyana* only and its concentration varied from 1.2 to 6.9% of the oil. The monoterpene 4-terpineol was found in four accessions of *A. tridentata* var. *vasseyana* and also in *A. longifolia* and *A. cana*. The concentration of 4-terpineole varied from 0.9 to 1.4% in the oil of *A. tridentata* var. *vasseyana*, from 1.7 to 2.5% in the oil of *A. longifolia*, and 1.5–2.1% in the oil of *A. cana*. Santolina triene was found in two accessions of *A. tridentata* var. *vasseyana* (#214, 215), and also in the oils of *A. tridentata* var. *tridentata*, *A. logifolia*, and *A. cana*. The concentration of santolina triene was 1.7–2.5% in the oil of *A. tridentata*, 3.5–3.7% in *A. cana*, and varied from 2.7 to 6.8% in the oil of *A. longifolia*. 

Arthole was found in the oils of the same two accession of *A. tridentata vasseyana*, in *A. tridentata* var. *tridentata*, in *A. tridentata* var. *wyomingensis*, and in the oils of *A. longifolia* and *A. cana*. The concentration of arthole was 10.3 and 13.0% in the oils of *A. tridentata* var. *vasseyana*, 3.8% in the oil of *A. tridentata* var. *tridentata*, 20.1% in the oil of *A. tridentata* var. *wyomingensis*, from 3.2 to 9.7% in the oil of *A. longifolia*, and 6.2 and 9.0% in the oil of *A. cana* (Table 5). 

Gama-terpinene was found in the oil of two accessions of *A. tridentata* var. *vasseyana* (at 1.0 and 2.2%), in *A. longifolia* (1.5–2.5% range) and in *A. cana* (1.7–1.8%) (Table 6). Alpha-santoline alcohol was found in the same two accessions of *A. tridentata* var. *vasseyana* (at 3.7 and 5.9%), in two accessions of *A. longifolia* (at 6.0 and 7.6%), and in one accession of *A. cana* (at 11.3%). Chrysanthemyl alcohol was found only in the same two accessions of *A. tridentata* var. *vasseyana* (at 4.4 and 11.4%). Beta-pinene was only found in the oil of one accession of *A. tridentata* var. *tridentata* at 2.2% concentration (Table 6). 

The following more significant oil constituents were only found in one or two accessions of *A. tridentata* var. *vasseyana* and did not vary within the accessions: periritenone (average concentration of 4.1%) in three accessions, artemisyl acetate (2.9%) in one accession, myrtenol (at 1.2%) in two accessions, borneol/lavandulol (1.9%) in two accessions, *trans*-arbusculone (1.9%) in one accession, piperitenone/citronellyl acetate (at 7.3%) in two accessions (Table 7).

### 2.5. Antioxidant Capacity of Artemisia Species and Subspecies

The antioxidant capacity of the EOs varied between the species and subspecies, with the highest being 80.5 μmol/g for *A. tridentata* var. *wyomingensis* and 60.5 μmol/g in *A. tridentata* var. *tridentata*; the antioxidant capacity of the rest of the oils was not different from the antioxidant capacity of the oils from the above two species (Table 8). This is the first report on antioxidant activity of the EO of the sagebrush species tested in this study; however, there is a previous report on *A. tridentata* var. *wyomingensis* leaf extract (70% ethanol extraction for 24 h) [42], but not on sagebrush EO. 

None of the accessions contained any podophyllotoxin, indicating that *Artemisia* species and subspecies collected in this study did not contain podophyllotoxin. 

### 2.6. Antileishmanial Evaluations

Compounds from some other *Artemisia* species (e.g., the endoperoxide artemisinin from *A. annua*) were reported to possess leishmanicidal activity [43]. Compounds extracted from *A. aucheri* have shown antileishmanial effect [44]. Ethanol extract from *A. absinthium* was found to be effective against *Leishmania major* L. in vitro [45]. The essential oil of *A. absinthium* was also reported to have promise as active compounds source against *Leishmania* [46].

In this study, representative essential oil samples from each of the species *A. cana* Pursh var. *cana*, *A. longifolia*, *A. tridentata* Nutt. var. *tridentata*, *A. tridentata* Nutt. var. *vaseyana*, and *A. tridentata* Nutt. var. *wyomingensis* were each evaluated against *Leishmania donovani* promastigotes and none demonstrated activity above 50% inhibition when evaluated at 80 μg/mL (Table S11). The bioassays used in this study are suited for the discovery of new therapeutic agents and hence the concentrations used in the analysis were much lower than those in previous studies. 

### 2.7. Antiplasmodial Evaluations

Representative essential oil samples from each of the species *A. cana* Pursh var. *cana*, *A. longifolia*, *A. tridentata* Nutt. var. *tridentata, A. tridentata* Nutt. var. *vaseyana*, and *A. tridentata* Nutt. var. *wyomingensis* were evaluated against *Plasmodium falciparum* D6 at 15,867 ng/mL and none of them demonstrated activity above 50% inhibition to warrant secondary evaluations for LC_50_ determinations (Table S11). 

### 2.8. Antimicrobial Evaluations 

Representative essential oil samples from each of the species *A. cana* Pursh var. *cana*, *A. longifolia*, *A. tridentata* Nutt. var. *tridentata*, *A. tridentata* Nutt. var. *vaseyana*, and *A. tridentata* Nutt. var. *wyomingensis* were evaluated against *Candida albicans*, *Candida glabrata*, *Candida krusei*, *Aspergillus fumigatus, Cryptococcus neoformans, Staphylococcus aureus*, Methicillin-resistant *S. aureus, Escherichia coli, Pseudomonas aeruginosa*, and *Mycobacterium intracellulare*, but none of them demonstrated activity above 50% inhibition when evaluated at 50 μg/mL (Table S12).

## 3. Materials and Methods

### 3.1. Collection of the Plant Material 

Two separate collections of sagebrush species were conducted and included in this study. In 2011, we conducted a comprehensive study in the Bighorn Mountains area to identify the species of sagebrush and analyze their EO content, profile, and bioactivity. Accessions were collected from a total of 22 sites at various elevations (1150–2988 m a.s.l.) (Table 1). In a separate study conducted in 2014, additional accessions were collected from 12 sites at elevations ranging from 1108 to 2902 m a.s.l. (Table 1). The sagebrush aboveground herbage was collected from each collection site, and the GPS coordinates were recorded (Table 1). Plant material from each collection site was identified by Ms. Bonnie Heidel at the Rocky Mountain Herbarium, at the University of Wyoming (http://www.uwyo.edu/wyndd/about-wyndd/staff/bonnie-heidel.html), accessed on 28 April 2022.

The majority of the collected accessions belonged to the big sagebrush subspecies: *A. tridentata* Natt. var. *vaseyana* (Rydb.) Beetle (mountain big sagebrush), *A. tridentata* Natt. var. *wyomingensis* Beetle and Young (Wyoming big sagebrush), and *A. tridentata* Natt. var. *tridentata* (basin big sagebrush). The most frequent and widespread species was *A. tridentata* Natt. var. *vaseyana* (Rydb.). In addition, we collected and identified the following species in the area: *Artemisia cana* Pursh (silver sagebrush), *A. ludoviciana* Nutt. (white sagebrush), and *A. longifolia* Nutt. (longleaf wormwood).

### 3.2. Essential Oil Extraction

#### 3.2.1. Steam Distillation 

Subsamples of *Artemisia* species and subspecies collected in 2011 were subjected to steam distillation for extraction of the EO. Representative samples of 500 g fresh material that included all aboveground plant parts, leaves, inflorescences, and annual stems not thicker than 2 mm were cut into approximately 2.5 cm long pieces. Then, each sample was immediately placed into the bioflask of 2 L steam distillation units (HeartMagic, Rancho Santa Fe, CA, USA) and steam distilled for 60 min as described previously for spearmint and peppermint [47,48].

#### 3.2.2. Hydro-Distillation

Essential oil from the accession samples collected in 2014 was extracted via hydro-distillation. A 100 g fresh plant biomass sample consisting of stems and leaves only or inflorescences only, cut into approximately 2.5 cm long pieces, was placed into a 2 L boiling flask along with 1.5 L water and distilled for 60 min. 

Distillation time was measured from when the first drop of EO appeared in the glass Florentine vessel (the separator), and at the end of the 60 min period, the power was shut down and the distillation discontinued. After the distillation of each sample, the water was drained from the Florentine vessel and the oil was collected in a glass vial and placed in a freezer. The collected EOs were separated from the remaining water, measured on an analytical scale, and the oil content (oil yield) was calculated as grams of oil per 100 g of fresh aboveground sagebrush biomass. Afterward, the EO samples were stored in at −5 °C in a freezer until the oils were analyzed for chemical profile on gas chromatography-mass spectrometry (GC-MS). 

### 3.3. Gas Chromatography (GC) Mass Spectroscopy (MS) of the Sagebrush Species Essential Oil (EO)

The GC-MS analysis of the sagebrush EO samples was conducted as described previously [49]. Briefly, the sagebrush EO samples (all samples in three replicates) were analyzed on a GC-MS instrument (Hewlett Packard Model 6890; Hewlett-Packard, Palo Alto, CA, USA). The carrier gas was helium at a mean speed of 40 cm/s^−1^, at 11.7 psi (60 °C), and a constant flow rate at 2.5 mL/min^−1^. The injection was split 60:1, 0.5 µL, with 220 °C injector temperature. The GC oven temperature program was as described previously: 60 °C for 1 min and 10 °C/min to 250 °C. The column was HP-INNOWAX (crosslinked polyethylene glycol; 30 m × 0.32 mm × 0.5 mm), and the flame ionization detector temperature was 275 °C. The sagebrush EO constituents were expressed as percentage of all the constituents in the EO. The identification of individual constituent peaks was completed using standard compounds, through retention index values and through MS spectra comparison. 

### 3.4. Podophyllotoxin Extraction and Measurements 

The *Artemisia* samples collected in 2011 were also subjected to chemical analyses for podophyllotoxin. The podophyllotoxin extraction and purification followed a previously described procedures [50] and as described in a podophyllotoxin analyses of *Juniperus* paper [51]. 

### 3.5. Antimicrobial, Antimalarial, and Antileishmanial Activity and Cytotoxicity 

The EO from the accessions collected in 2011 were submitted for analyses of antimicrobial, antimalarial, and antileishmanial activity and cytotoxicity. The assays were conducted at The University of Mississippi, National Center for Natural Products Research using methods developed at the Center and as described previously [51].

### 3.6. Antioxidant Activity of the EOs of Artemisia Species and Subspecies from This Study 

The antioxidative capacity of the *Artemisia* oils collected in 2011 was determined by the oxygen radical absorbance capacity (ORAC) [52,53] and as described previously [54]. All samples were taken in triplicate.

### 3.7. Statistical Analyses of the Data 

Analysis of variance was completed for EO yield, antioxidant activity, and the concentrations of α-pinene, camphene, eucalyptol, camphor, trans-α-necrodol-acetate, fragranol, grandisol, piperitenone/citronellyl acetate, borneol, trans-pinocarveol, cis-arbusculone, pinocarvone, 4-terpineol, myrtenol, santolina triene, borneol/lavandulol, arthole, gamma-terpinene, α-santoline alcohol, chrysanthemyl alcohol, beta-pinene, and chrysanthenone using either a nested design or a completely randomized design model, and both designs used 3 replications. A nested design (species, and subspecies nested within species effects in the model) was used for EO yield, and the constituents obtained from more than one species. However, for the constituents obtained only within a species, a completely randomized design was used to compare the subspecies. The analysis was completed using the Mixed Procedure of SAS [54], and for each response, the validity of model assumptions was verified by examining the residuals as described in [55]. Since the effects of species (where applicable) and subspecies were significant (with the exception of piperitenone/citronellyl acetate, myrtenol, and borneol/lavandulol where the subspecies were not significantly different), multiple means comparison of the subspecies was completed using Tukey’s multiple means comparison method at the 5% level of significance. 

## 4. Conclusions

This study investigated the EO composition and bioactivity of several sagebrush species in the Western United States, and the findings revealed wide variation in the EO yield and composition between the sagebrush species, as well as within a species or subspecies, partially confirming the hypothesis. These findings suggest the presence of chemotypes within some species of sagebrush. 

The outcomes from this study refuted part of our hypothesis that the essential oil obtained from the sagebrush species would have antileishmanial, antimalarial, and antimicrobial activities. The hypothesis was based on reports of such activities for extracts and derivatives from other *Artemisia* species, such as *A. annua*, *A. aucheri*, *A. absinthium*. Synthetic 1,2-dioxanes were shown to possess leishmanicidal activity, whilst the natural endoperoxide artemisinin was not effective against *L. donovani* [43]. Compounds extracted from *A. aucheri* have shown antileishmanial effects against *L. major* [44]. Ethanol extract from *A. absinthium* was found to be effective against *Leishmania major* L. in vitro [45]. The EO of *A. absinthium* was also reported to have promise as active compounds against *Leishmania* [46]. It is worth noting that the bioassays used in this study are suited to the discovery of new therapeutic agents and the concentrations used in the analysis are perhaps much lower than the concentrations used in previous reports. In addition, we used whole oils from different *Artemisia* species.

The EOs of the sagebrush species in this study did not show significant antimicrobial activities, which contradicts other reports [18]. Most probably, the differences between the antimicrobial activity of the tested EO in this study and literature reports were due to the following: (1) higher concentrations used in the previous reports; (2) different assays; (3) differences in chemical constituents of the EOs in this study vs. literature reports. Indeed, a report [18] found differences in antifungal activity between different populations of *A. tridentata*. The authors of the report explained these differences with compositional dissimilarities of secondary metabolites between the different populations with respect to antifungal activity. 

Overall, wild grown sagebrush species and subspecies seem to be a largely untapped resource for EO with interesting and possibly desirable composition. Some of the accessions have yielded EO with significant concentrations of compounds such as camphor, eucalyptol, cis-thujone, α-pinene, α-necrodol-acetate, fragranol, grandisol, para-cymene, and arthole among others. Therefore, chemotypes can be selected and possibly introduced into culture and be grown for commercial production of these compounds. 

The results of our studies suggest immense chemical diversity exists that presents an opportunity for the selection of chemotypes/varieties with high concentration of EO with desirable composition.

## Figures and Tables

**Table 1:** Latin name, collection date, elevation and GPS coordinates for sagebrush accessions collected from Bighorn Mountains in Wyoming in fall of 2011 and fall of 2014.

Accession	Latin Name	Collection Date	Elevation, m	GPS Coordinates
201	*A. tridentata* Nutt. var. *vaseyana* (Rydb.) Boivin	24 August 2011	2383	−
202	*A. tridentata* Nutt. var. *vaseyana* (Rydb.) Boivin	24 August 2011	2406	44.8048, −107.5413
203	*A. tridentata* Nutt. var. *vaseyana* (Rydb.) Boivin	24 August 2011	2559	44.8126, −107.6095
204	*A. tridentata* Nutt. var. *vaseyana* (Rydb.) Boivin	24 August 2011	2377	44.7888, −107.9297
205	*A. tridentata* Nutt. var. *vaseyana* (Rydb.) Boivin	26 August 2011	2980	44.749, −107.7471
206	*A. tridentata* Nutt. var. *vaseyana* (Rydb.) Boivin	26 August 2011	2825	44.7588, −107.7556
207	*A. tridentata* Nutt. var. *vaseyana* (Rydb.) Boivin	26 August 2011	1907	44.7822, −107.968
209	*A. tridentata* Nutt. var. *vaseyana* (Rydb.) Boivin	23 September 2011	2253	44.3156, −106.9416
210	*A. tridentata* Nutt. var. *vaseyana* (Rydb.) Boivin	23 September 2011	2396	44.2512, −106.9562
211	*A. tridentata* Nutt. var. *vaseyana* (Rydb.) Boivin	23 September 2011	2423	44.1571, −107.2517
212	*A. tridentata* Nutt. var. *vaseyana* (Rydb.) Boivin	23 September 2011	2221	44.1325, −107.2526
213	*A. ludoviciana* Nutt. ssp. *ludoviciana*	26 September 2011	1643	44.6327, −107.0786
214	*A. tridentata* Nutt. var. *vaseyana* (Rydb.) Boivin	26 September 2011	2157	44.619, −107.1014
215	*A. tridentata* Nutt. var. *vaseyana* (Rydb.) Boivin	26 September 2011	2164	44.6186, −107.1102
216	*A. longifolia* Nutt.	27 September 2011	1166	44.8117, −106.9116
217	*A. longifolia* Nutt.	27 September 2011	1147	44.8354, −106.8735
218	*A. longifolia* Nutt.	27 September 2011	1149	44.8376, −106.8404
219	*A. cana* Pursh var. *cana*	27 September 2011	1292	44.8233, −107.2269
220	*A. cana* Pursh var. *cana*	27 September 2011	1333	44.826, −107.236
221	*A. tridentata* Nutt. var. *tridentata*	30 September 2011	2299	44.569, −107.5337
222	*A. tridentata* Nutt. var. *tridentata*	30 September 2011	2141	44.5742, −107.5666
223	*A. tridentata* Nutt. var. *wyomingensis* (Beetle and Young) Welsh	30 September 2011	1453	44.0279, −107.5646
250	*Artemisia* ssp.	28 October 2014	1166	44.8318, −106.8314
251	*A. tridentata* Nutt. var. *wyomingensis* (Beetle and Young) Welsh	28 October 2014	1192	44.8318, −106.8338
252	*A. cana* Pursh var. *cana*	28 October 2014	1128	44.8376, −106.8403
253	*A. cana* Pursh var. *cana*	28 October 2014	1126	44.8433, −106.8407
254	*A. cana* Pursh var. *cana*	28 October 2014	1136	44.8462, −106.8395
255	*A. cana* Pursh var. *cana*	29 October 2014	1118	44.8925, −107.0287
256	*A. cana* Pursh var. *cana*	29 October 2014	1119	44.8886, −107.0293
257	*A. cana* Pursh var. *cana*	29 October 2014	1130	44.8887, −107.0327
258	*A. cana* Pursh var. *cana*	29 October 2014	1105	44.8967, −107.0289
259	*A. tridentata* Nutt. var. *vaseyana* (Rydb.) Boivin	30 October 2014	2172	44.618, −107.1121
260	*A. tridentata* Nutt. var. *wyomingensis* (Beetle and Young) Welsh	30 October 2014	2524	44.7161, −107.4587
261	*A. tridentata* Nutt. var. *vaseyana* (Rydb.) Boivin	30 October 2014	2300	44.5691, −107.5338
262	*A. tridentata* Nutt. var. *vaseyana* (Rydb.) Boivin	30 October 2014	2895	44.6653, −107.5451

**Table 2:** Mean composition of alpha-pinene, camphene, eucalyptol and camphor (all in %) from six species and corresponding accessions. Blank cells indicate the constituents were not obtained from these accessions. *A. tridentata va* = *A. tridentata* Nutt. var. *vaseyana*; *A. tridentata tr* = *A. tridentata* Nutt. var. *tridentata*; *A. tridentata wy* = *A. tridentata* Nutt. var. *wyomingensis*.

Species	Accession	Alpha-Pinene	Camphene	Eucalyptol	Camphor
*A. tridentata va*	201	3.8 def	8.5 de	1.1 i	31.9 bcde
*A. tridentata va*	202	1.9 ef	6.7 e	2.4 i	27.3 de
*A. tridentata va*	203	0.9 f	0.1 g	0.8 i	0.3 h
*A. tridentata va*	204	2.9 def			
*A. tridentata va*	207	1.6 ef	8.4 de	14.8 e	29.3 cde
*A. tridentata va*	209	29.0 b	2.9 fg	12.3 g	1.8 gh
*A. tridentata va*	210	35.5 a	2.2 g	12.8 fg	0.6 h
*A. tridentata va*	211	5.3 d	13.8 b	17.9 d	44.8 a
*A. tridentata va*	212	5.6 d	13.6 bc	14.1 ef	45.4 a
*A. tridentata va*	214	1.3 ef	6.5 e	9.7 h	13.5 fg
*A. tridentata va*	215	23.5 c	2.1 g	2.5 i	3.2 gh
*A. tridentata tr*	221	3.2 def	10.5 cd	13.6 efg	43.2 ab
*A. tridentata tr*	222	4.3 de	21.5 a	21.2 c	41.3 ab
*A. tridentata wy*	223	1.1 ef	6.6 e	12.5 fg	21.6 ef
*A. tridentata wy*	216	2.8 def	5.8 ef	30.8 a	24.2 def
*A. longifolia*	217	2.8 def	7.0 e	24.6 b	27.7 de
*A. longifolia*	218	3.2 def	6.9 e	25.5 b	43.4 ab
*A. longifolia*	219	2.7 def	7.4 de	15.3 e	35.2 abcd
*A. cana*	220	3.2 def	6.6 e	20.5 c	40.6 abc
*A. cana*	213	1.9 ef	13.3 bc	17.9 d	46.2 a

Within each column, means followed by the same letter are not significantly different at the 5% level using Tukey’s method.

**Table 3:** Square root of mean squares error (MSE) that estimates the common standard deviation (σ).

Variable	$\sqrt{MSE}$
EO yield	0.073
antioxidant activity	6.71
α-pinene	0.949
camphene	1.02
eucalyptol	0.601
camphor	3.66
trans-α-necrodol-acetate	2.06
fragranol	2.43
grandisol	3.10
piperitenone/citronellyl acetate	1.04
borneol	0.154
trans-pinocarveol	1.14
cis-arbusculone	0.158
pinocarvone	0.420
4-terpineol	0.048
Myrtenol	0.123
santolina triene	0.304
borneol/lavandulol	0.070
arthole	1.09
gamma-terpinene	0.063
α-santoline alcohol	0.319
chrysanthemyl alcohol	1.06
beta-pinene	0.956
chrysanthenone	4.17

**Table 4:** Mean trans-α-necrodol-acetate, fragranol, grandisol, cis-arbusculone, trans-pinocarveol and chrysanthenone (all in %) from 10 collections of the *A. tridentata* Nutt. var. *vaseyana* (Rydb.) Boivin. Blank cells indicate the constituents were not obtained from these accessions. *A. tridentata va* = *A. tridentata* Nutt. var. *vaseyana*.

Species	Accession Number	Trans-α-Necrodol-Acetate	Fragranol	Grandisol	Cis-Arbusculone	Trans-Pinocarveol	Chrysanthenone
*A. tridentata va*	201	5.7 e	5.7 c	6.5 d			21.61 a
*A. tridentata va*	202	13.5 d	12.4 bc	17.6 c			1.21 b
*A. tridentata va*	203	25.9 c	20.3 a	36.2 a			
*A. tridentata va*	205	34.8 b	14.9 ab	31.1 ab			
*A. tridentata va*	206	45.1 a	15.0 ab	26.2 bc			
*A. tridentata va*	207						11.86 ab
*A. tridentata va*	209				3.1 a	24.8 a	
*A. tridentata va*	210				2.3 b	20.9 b	
*A. tridentata va*	214	8.2 de					
*A. tridentata va*	215	4.8 e				6.0 c	

Within each column, means followed by the same letter are not significantly different at the 5% level using Tukey’s method.

**Table 5:** Mean borneol, pinocarvone, 4-terpineol, santolina triene and arthole (all in %) from six species and their corresponding collection number. Blank cells indicate the constituents were not obtained from these accessions. *A. tridentata va* = *A. tridentata* Nutt. var. *vaseyana*; *A. tridentata tr* = *A. tridentata* Nutt. var. *tridentata*; *A. tridentata wy* = *A. tridentata* Nutt. var. *wyomingensis*.

Species	Accession Number	Borneol	Pinocarvone	4-Terpineol	Santolina Triene	Arthole
*A. tridentata va*	209		5.3 b	1.3 e		
*A. tridentata va*	210		6.9 a	1.4 de		
*A. tridentata va*	211		1.0 c	1.1 f		
*A. tridentata va*	212		1.2 c	0.9 g		
*A. tridentata va*	214		8.1 a		2.5 cde	13.0 b
*A. tridentata va*	215		1.9 c		1.8 de	10.3 c
*A. tridentata tr*	221				1.7 e	3.8 f
*A. tridentata wy*	223					20.1 a
*A. longifolia*	216	2.3 c		2.5 a	2.7 cd	9.7 cd
*A. longifolia*	217	2.4 bc		1.7 c	6.8 a	6.8 e
*A. longifolia*	218	2.4 bc		2.2 b	3.1 bc	3.2 f
*A. cana*	219	2.3 c		1.5 d	3.5 b	9.0 d
*A. cana*	220	2.6 b		2.1 b	3.7 b	6.2 e
*A. ludoviciana*	213	4.6 a		1.3 e	2.2 de	

Within each column, means followed by the same letter are not significantly different at the 5% level using Tukey’s method.

**Table 6:** Mean gamma-terpinene, alpha-santoline alcohol, chrysanthemyl alcohol and beta-pinene (all in %) from four species and their subspecies. Blank cells indicate the constituents were not obtained from these accessions. *A. tridentata va* = *A. tridentata* Nutt. var. *vaseyana*; *A. tridentata tr* = *A. tridentata* Nutt. var. *tridentata*.

Species	Accession Number	Gamma- Terpinene	Alpha- Santoline Alcohol	Chrysanthemyl Alcohol	Beta-Pinene
*A. tridentata va*	204				7.23 a
*A. tridentata va*	214	1.0 e	3.7 d	11.37 a	
*A. tridentata va*	215	2.2 b	5.9 c	4.44 b	
*A. tridentata tr*	222				2.21 b
*A. longifolia*	216	1.5 d	7.6 b		
*A. longifolia*	217	2.5 a	6.0 c		
*A. longifolia*	218	1.5 d			
*A. cana*	219	1.8 c	11.3 a		
*A. cana*	220	1.7 cd			

Within each column, means followed by the same letter are not significantly different at the 5% level using Tukey’s method.

**Table 7:** Piperitenone/citronellyl acetate, artemisyl acetae, myrtenol, borneol/lavandulol, trans-rbusculone, sabinene, alpha-phellendrene, para-cymene and *trans*-ocimene that were obtained from only one accession (shown in bracket in Column 1) or without significant difference among accessions.

Species (Accession Numbers)	Constituent	Mean (%)
*A. tridentata* Nutt. var. *vaseyana* (201, 202, 203)	Piperitenone/citronellyl acetate	4.13
*A. tridentata* Nutt. var. *vaseyana* (209)	Artemisyl acetae	2.88
*A. tridentata* Nutt. var. *vaseyana* (209, 210)	Myrtenol	1.2
*A. tridentata* Nutt. var. *vaseyana* (211, 212)	Borneol/Lavandulol	1.9
*A. tridentata* Nutt. var. *vaseyana* (215)	Trans-arbusculone	1.88
*A. tridentata* Nutt. var. *vaseyana* (214, 215)	Piperitenone/Citronellyl acetate	7.26
*A. tridentata* Nutt. var. *vaseyana* (204)	Sabinene	12.32
*A. tridentata* Nutt. var. *vaseyana* (204)	Alpha-phellendrene	16.86
*A. tridentata* Nutt. var. *vaseyana* (204)	Para-cymene	31.83
*A. tridentata* Nutt. var. *vaseyana* (204)	Trans-ocimene	0.94

**Table 8:** Mean antioxidant activity (µM Trolox equivalents/g) of the essential oils from the five species.

Species	Accession Number	Antioxidant Activity
*A. tridentata* Nutt. var. *vaseyana*	207	63.1 ab
*A. tridentata* Nutt. var. *tridentata*	221	60.5 b
*A. tridentata* Nutt. var. *wyomingensis*	223	80.5 a
*A. longifolia*	216	73.4 ab
*A. cana*	220	71.5 ab

Means followed by the same letter are not significantly different at the 5% level using Tukey’s method.

## Data Availability

Data are contained within the article.

## Supplementary Materials

The following are available online at https://www.mdpi.com/article/10.3390/plants11091228/s1, Table S1: Mean (using *n* = 3) content (%) of constituents in essential oil obtained by steam distillation of *A. tridentata* Nutt. var. *vaseyana* (Rydb.) Boivin accessions biomass collected from Bighorn Mountains in Wyoming; Table S2: Mean (using *n* = 3) content (%) of constituents in essential oil obtained by steam distillation of *A. tridentata* Nutt. var. *tridentata* accessions biomass collected from Bighorn Mountains in Wyoming; Table S3: Mean (using *n* = 3) content (%) of constituents in essential oil obtained by steam distillation *A. tridentata* Nutt. var. *wyomingensis* (Beetle and Young) Welsh accessions biomass collected from Bighorn Mountains in Wyoming; Table S4: Mean (using *n* = 3) content (%) of constituents in essential oil obtained by steam distillation of *A. cana* Pursh var. *cana* accessions biomass collected from Bighorn Mountains in Wyoming; Table S5: Mean (using *n* = 3) content (%) of constituents in essential oil obtained by steam distillation of *A. longifolia* Nutt. accessions biomass collected from Bighorn Mountains in Wyoming; Table S6: Mean (using *n* = 3) content (%) of constituents in essential oil obtained by steam distillation of *A. ludoviciana* Nutt. ssp. *ludoviciana* accessions biomass collected from Bighorn Mountains in Wyoming; Table S7: Mean (using *n* = 3) content (%) of constituents in essential oil obtained by hydrodistillation of *A. tridentata* var. *vaseyana* accessions biomass (leaves) collected from Bighorn Mountains in Wyoming; Table S8: Mean (using *n* = 3) content (%) of constituents in essential oil obtained by hydrodistillation of *A. tridentata* var. *wyomingensis* accessions biomass (leaves or inflorescences) collected from Bighorn Mountains in Wyoming; Table S9: Mean (using *n* = 3) content (%) of constituents in essential oil obtained by hydrodistillation of *A. cana* var. *cana* accessions biomass (leaves or inflorescences) collected from Bighorn Mountains in Wyoming; Table S10: Mean (using *n* = 3) content (%) and range of EO obtained by hydrodistillation for accessions from Bighorn Mountains in Wyoming in the Fall of 2014; Table S11. Evaluation of select essential oils against *Leishmania donovani* and *Plasmodium falciparum* D6. Data are percent inhibition. Primary evaluations performed at 80 ug/mL for *L. donovani* and 15,867 ng/mL for *P. falciparum* D6; Table S12. Evaluation of select essential oils against opportunistic infection pathogens. Primary evaluations performed at 50 ug/mL and data are percent inhibition.
