# Supplementary material for: Essential Oil Yield, Composition, and Bioactivity of Sagebrush Species in the Bighorn Mountains
Source: Plants (Basel). 2022 May 1;11(9):1228. doi: 10.3390/plants11091228 (PMC9103225; doi:10.3390/plants11091228)
Supplement: Supplementary file 1 [file plants-11-01228-s001.zip › plants-1686323-supplementary.pdf]

plants

Article

Essential Oil Yield, Composition, and Bioactivity of Sagebrush Species in the Bighorn Mountains

Valtcho D. Zheljazkov [1,*], Charles L. Cantrell [2], Ekaterina A. Jeliazkova [1], Tess Astatkie [3], and Vicki Schlegel [4]

[1] Crop and Soil Science Department, 3050 SW Campus Way, Oregon State University, Corvallis, OR 97331, USA
[2] Natural Products Utilization Research, USDA-Agricultural Research Service, University of Mississippi, University, MS 38677, USA; charles.cantrell@usda.gov
[3] Faculty of Agriculture, Dalhousie University, Truro, NS B2N 5E3, Canada; astatkie@dal.ca
[4] University of Nebraska–Lincoln, Department of Food Science and Technology, Department of Food Science and Technology, 326 Food Technology Complex, Lincoln, NE 68583, USA; vschlegel3@unl.edu
* Correspondence: valtcho.jeliazkov@oregonstate.edu

Supplemental tables

Table S1. Mean (using n = 3) content (%) of constituents in essential oil obtained by steam distillation of *A. tridentata* Nutt. var. *vaseyana* (Rydb.) Boivin accessions biomass collected from Bighorn Mountains in Wyoming.

**Constituent**	**RT(mins)**	**Accessions**											
		**#201**	**#202**	**#203**	**#204**	**#205**	**#206**	**#207**	**#209**	**#210**	**#211**	**#212**	**#214**
1-Heptene	2.55	-	-	-	0.63	-	-	-	-	-	-	-	-
1-Octene	4.76	-	-	-	0.21	-	-	0.04	-	-	-	-	-
Hexanal	4.96	-	0.03	-	-	-	-	-	-	-	-	-	-
Ethyl isovalerate/Hexanol	6.92	-	-	-	0.02	-	-	-	-	-	-	-	-
**1-Nonene**	8.30	-	-	-	**2.48**	-	-	-	-	-	-	-	-
2-Acetylfuran	8.69	-	-	-	-	-	-	0.09	-	-	-	-	-
**Santolina triene**	8.92	0.10*	0.33	-	-	-	-	**2.51**	-	0.40	0.18	0.24	2.46
Tricyclene	9.49	0.33	0.31	-	-	-	-	0.42	-	-	0.65	0.64	-
Alpha-thujene	9.69	-	-	-	0.19	-	-	0.08	-	0.14	-	-	-
**Alpha-pinene**	9.96	**2.98**	**2.92**	0.94	**2.94**	0.04	-	**1.62**	**29.0**	**35.5**	**5.27**	**5.65**	**1.34**
**Camphene**	10.54	**6.55**	**6.75**	0.11	-	0.21	-	**8.37**	**2.89**	**2.2**	**13.76**	**13.58**	**6.55**
Thuja-2,4(10)-diene	10.70	-	-	-	-	-	-	-	0.25	-	-	-	-
4,4-Dimethyl-2-butenolide/Thuja-2,4(10)-diene	10.79	-	-	-	-	-	-	0.05	-	-	-	-	-
Isobutyl butyrate	10.81	-	-	-	0.22	-	-	-	-	-	-	-	-
**Sabinene**	11.54	0.24	0.17	0.03	**12.32**	-	-	0.36	0.42	0.42	0.77	0.59	-
Arthole / Beta-pinene	11.68	-	0.67	-	-	-	-	-	-	-	-	-	-
**Arthole**	11.71	-	-	-	-	-	-	**6.91**	-	-	-	-	**12.97**
**Beta-pinene**	11.72	0.45	-	-	7.23	-	-	-	**1.93**	**2.68**	**2.34**	**1.72**	-
Dehydro-1,8-cineole	12.21	-	-	-	-	-	-	0.24	0.33	0.27	0.25	0.21	-
1,6-Dimethylhepta-1,3,5-triene	12.25	0.41	-	0.2	-	0.16	0.11	-	-	-	-	-	0.56
Dehydro-1,8-cineole / 1,6-dimethylhepta-1,3,5-triene	12.26	-	0.40	-	-	-	-	-	-	-	-	-	-
Mesitylene	12.32	0.09	-	-	-	-	-	0.06	-	-	-	-	-
**Myrcene**	12.36	-	-	-	**7.46**	-	-	-	-	-	-	-	-
Delta-3-carene	12.44	-	0.03	-	-	-	-	-	-	-	-	-	-
Butyl butanoate	12.45	-	-	-	0.12	-	-	-	-	-	-	-	-
Yomogi alcohol	12.54	0.09	0.09	0.10	-	0.13	0.20	0.08	0.22	-	-	-	0.17
**Alpha-phellendrene**	12.76	-	-	-	**16.86**	-	-	0.11	-	-	-	-	-
Ortho-methylanisole	13.05	-	-	-	0.06	-	-	-	-	-	-	-	-
Hexyl acetate	13.09	0.04	-	-	-	-	-	-	-	-	-	-	-
Alpha-terpinene	13.23	0.07	0.08	-	0.46	-	-	0.24	0.37	0.41	0.31	0.21	-
1,2,4-Trimethyl benzene	13.46	0.06	-	-	-	-	-	-	-	-	-	-	-
**Para-cymene**	13.52	0.12	0.09	0.06	**31.83**	-		0.54	0.54	0.53	0.47	0.51	0.37
**Limonene**	13.71	0.19	0.31	0.04	**3.30**	0.18	-	-	-	-	-	-	-
**Eucalyptol**	13.81	**1.23**	**2.35**	0.75	-	0.20	-	**14.81**	**12.27**	**12.82**	**17.94**	**14.14**	**9.72**
Cis-ocimene	14.13	-	-	-	0.10	-	-	-	-	-	-	-	-
Sorbyl acetate	14.15	0.10	0.08	0.09	-	0.09	0.05	-	-	-	-	-	-

Table S1 Continued. Mean (using n = 3) content (%) of constituents in essential oil obtained by steam distillation of *A. tridentata* Nutt. var. *vaseyana* (Rydb.) Boivin accessions biomass collected from Bighorn Mountains in Wyoming.

Constituent	RT(mins)	Accessions											
		#201	#202	#203	#204	#205	#206	#207	#209	#210	#211	#212	#214
**Trans-ocimene**	14.55	-	-	-	0.94	-	-	-	**3.05**	**2.30**	-	-	-
Cis-arbusculone	14.63	-	0.04	-	-	-	-	0.08	-	-	-	-	0.18
Santolina epoxide	14.70	-	-	-	-	-	-	0.29	-	-	-	-	-
Pentyl isobutanoate	14.90	-	-	-	0.16	-	-	-	-	-	-	-	-
Isoterpinolene / Isoterpinene	14.91	0.13	0.18	-	-	-	-	-	-	-	-	-	-
**Gamma-terpinene**	14.92	-	-	-	-	-	-	**1.34**	0.72	**1.01**	0.67	0.51	**1.02**
Isopentyl butanoate/ Gamma-terpinene	14.97	-	-	-	0.87	-	-	-	-	-	-	-	-
Bergamal	15.01	-	-	-	-	-	-	-	-	-	-	-	-
Cis-sabinene hydrate	15.26	-	-	-	-	-	-	-	0.19	0.30	-	-	0.22
**Trans-arbusculone**	15.44	-	0.06	-	-	-	-	-	**2.00**	**1.71**	-	-	0.89
Artemisia alcohol	15.87	-	-	-	-	-	-	-	0.16	-	-	-	0.20
Terpinolene	16.06	-	-	-	0.50	-	-	0.20	0.24	0.26	0.15	0.08	-
2,6-xylenol / Alpha-pinene oxiide	16.43	-	-	-	-	-	-	-	-	-	-	-	-
Aplha pinene oxide / 2,6-Xylenol	16.43	-	-	-	-	-	-	0.13	-	-	-	-	-
Linalool / Ipsenol	16.49	-	-	-	0.22	-	-	-	-	-	-	-	-
Nonanal	16.63	-	-	-	0.16	-	-	-	-	-	-	-	0.24
**Cis-thujone**	16.71	0.39	0.16	0.09	0.05	-	-	-	-	-	**4.26**	**7.50**	-
2,6-xylenol / Benzyl ethanol	16.90	0.12	0.06	-	-	-	-	-	-	-	-	-	-
Trans-thujone	17.10	-	-	-	0.05	-	-	-	-	-	0.35	0.57	-
**Chrysanthenone**	17.23	**17.97**	**1.21**	-	-	-	-	**11.86**	**1.54**	0.39	**2.29**	**1.37**	0.17
Methyl octanoate	17.39	-	-	-	0.14	-	-	-	-	-	-	-	-
Alpha-campholenal	17.50	-	-	-	0.04	-	-	-	-	-	-	-	0.40
Cis-para-mentha-2,8-dien-1-ol	17.72	-	-	-	-	-	-	-	-	-	0.17	0.24	-
1-terpineol	17.79	-	0.10	-	-	-	-	-	-	-	-	-	-
**Trans-pinocarveol**	18.00	-	**5.79**	0.08	0.06	-	-	-	**24.82**	**20.94**	-	-	0.24
**Camphor**	18.21	**24.91**	**23.43**	0.28	-	0.43	-	**29.32**	**1.76**	0.58	**44.85**	**45.37**	**13.51**
**Alpha-santoline alcohol**	18.28	-	-	-	-	-	-	**9.72**	-	-	-	-	**3.71**
Isobutyl hexanoate	18.36	-	-	-	0.19	-	-	-	-	-	-	-	-
**Trans-chrysanthemol**	18.85	**3.83**	**3.67**	**2.52**	-	**3.96**	**4.48**	-	-	-	-	-	-
**Chrysanthemyl alcohol**	18.91	-	-	-	-	-	-	0.76	-	-	-	-	11.37
**Pinocarvone**	18.91	-	0.83	0.25	-	-	-	0.62	**5.33**	**6.90**	**1.03**	**1.24**	**8.07**
**Borneol / lavandulol**	19.05	**1.35**	**1.11**	-	-	0.22	-	-	-	-	**1.85**	**1.93**	-
**Borneol**	19.10	-	-	-	-	-	-	**2.03**	0.44	0.28	-	-	**1.55**
Atremisyl acetate	19.21	-	-	-	-	-	-	-	-	-	-	-	-
(3E,5Z)-1.3.5-undecatriene	19.24	-	-	-	0.24	-	-	-	-	-	-	-	-
Artemisyl acetae	19.31	-	-	-	-	-	-	-	**2.88**	-	-	-	-
Hexyl butanoate	19.88	-	-	-	0.24	-	-	-	-	-	-	-	-
Alpha-terpineol	19.98	-	-	-	-	-	-	-	-	-	0.25	0.10	-
Myrtenal / Methyl chavicol	20.15	-	-	-	0.24	-	-	-	-	-	-	-	-

Table S1 Continued. Mean (using n = 3) content (%) of constituents in essential oil obtained by steam distillation of *A. tridentata* Nutt. var. *vaseyana* (Rydb.) Boivin accessions biomass collected from Bighorn Mountains in Wyoming.

Constituent	RT(mins)	Accessions											
		#201	#202	#203	#204	#205	#206	#207	#209	#210	#211	#212	#214
Myrtenal / Myrtenol	20.19	-	-	-	-	-	-	-	-	-	-	-	0.80
Cis-dihyrocarvone/Myrtenol/Myrtenal	20.20	-	-	-	-	-	-	0.61	-	-	-	-	-
Myrtenal	20.21	-	-	-	-	-	-	-	-	-	0.71	0.66	-
**Myrtenol**	20.23	-	-	-	-	-	-	-	**1.22**	**1.24**	-	-	-
Decanal	20.37	-	-	-	0.30	-	-	-	-	-	-	-	-
Bornyl acetate	20.39	-	-	-	-	-	-	0.49	-	-	0.44	0.39	0.33
**Fragranol**	21.01	**7.48**	**12.37**	**20.29**	-	**14.88**	**15.00**	-	-	-	-	-	-
**Trans-ethyl-chrysanthemumate / Trans-alpha-necrodol-acetate**	23.33	**10.24**	**13.48**	**25.91**	-	**34.78**	**45.15**	-	-	-	-	-	**8.17**
Isobornyl acetate	23.61	-	-	0.59	-	-	-	-	-	-	-	-	-
Lavandulyl acetate	23.65	0.39	-	0.27	-	0.27	0.91	-	-	-	-	-	0.21
Trans-pinocaryl acetate	23.81	-	-	-	-	-	-	-	0.26	0.27	-	-	-
3-thujanol acetate / Cis-alpha-necrodol acetate	24.22	-	-	-	-	0.25	-	-	-	-	-	-	-
**Grandisol**	25.14	**8.78**	**17.64**	**36.18**	-	**31.08**	**26.18**	-	-	-	-	-	-
Alpha-cubebene	25.77	-	-	-	-	-	-	-	-	-	-	-	-
Beta-elemene	26.08	-	-	-	0.38	-	-	-	-	-	-	-	-
**Piperitenone / Citronellyl acetate**	26.35	**5.74**	**4.59**	**4.43**	-	**4.75**	**3.2**	-	-	-	-	-	**6.60**
**Beta-caryophyllene**	26.64	0.53	0.74	-	**1.27**	0.98	0.48	-	0.34	0.27	-	-	0.42
Linalool isovalerate	26.96	0.82	0.85	2.64	-	2.37	0.85	-	-	-	-	-	-
2-phenyl-ethyl butanoate	26.97	-	-	-	0.44	-	-	-	-	-	-	-	-
Cis-beta-farnesene	27.20	-	-	-	0.35	-	-	-	-	-	-	-	-
Gamma-curcumeme	27.64	0.38	0.74	0.81	-	0.64	0.55	-	0.39	0.36	-	-	-
**Curcumene ar / Germacrene D**	27.73	**1.04**	0.96	0.74	-	0.53	0.24	-	-	-	-	-	-
Germacrene D	27.78	-	-	-	1.06	-	-	-	0.75	0.76	-	-	0.54
**Davana ether isomer**	27.81	-	-	-	-	-	-	-	**1.14**	**1.73**	-	-	-
Beta-selinene	27.87	-	-	-	0.11	-	-	-	-	-	-	-	-
Bicyclogermacrene	28.03	-	-	-	-	-	-	-	0.26	-	-	-	-
Germacrene A	28.03	-	-	-	0.38	-	-	-	-	-	-	-	-
Delta-cadinene	28.39	-	-	-	0.48	-	-	-	-	-	-	-	-
Cis-3-hexenyl benzoate	29.04	-	-	-	0.50	-	-	-	-	-	-	-	-
Trans-2-hexenyl benzoate	29.21	-	-	-	0.43	-	-	-	-	-	-	-	-
Caryophyllene oxide	29.36	-	-	-	-	-	-	-	-	-	-	-	-
Davanone B	29.54	-	-	-	-	-	-	-	0.43	0.41	-	-	-
Davanone	29.61	-	-	-	-	-	-	-	0.65	0.62	-	-	-
Beta-oplopenone	29.68	-	-	-	0.36	-	-	-	-	-	-	-	-
Davanol D1 isomer	29.95	-	-	-		-	-	-	0.77	0.61	-	-	-
Davanol D2 isomer	30.48	-	-	-		-	-	-	0.27	0.26	-	-	-
Anthracene-1,2,3,4,5,6,7,8-octahydro-9,10-dimethyl	32.04	-	-	-	0.96	-	-	-	-	-	-	-	-

Pyran-5-one(2,2-dimethyl-7-isobutyl-2H,5H-pyrano(4,3-b)-)	32.95	-	-	-	-	0.28	-	-	-	-	-	-	-

Table S2. Mean (using n = 3) content (%) of constituents in essential oil obtained by steam distillation of *A. tridentata* Nutt. var. *tridentata* accessions biomass collected from Bighorn Mountains in Wyoming.

Constituent	RT (mins)	Accessions	
		#221	#222
**Santolina triene**	8.95	**1.68**	-
Tricyclene	9.50	0.51	1.02
**Alpha-pinene**	10.02	**3.23**	**4.32**
**Camphene**	10.68	**10.48**	**21.54**
Sabinene	11.56	0.40	0.62
**Arthole**	11.70	**3.84**	-
**Beta-pinene**	11.73	-	**2.21**
Dehydro-1,8-cineole	12.22	0.30	0.25
Alpha-terpinene	13.25	0.19	0.33
Para-cymene	13.58	0.38	0.25
Limonene	13.71	-	0.37
**Eucalyptol**	13.99	**13.62**	**21.18**
Cis-arbusculone	14.64	0.25	-
Gamma-terpinene	14.92	0.53	0.67
Artemisia ketone	14.99	0.52	-
Cis-sabinene hydrate	15.25	0.39	0.26
Cis-thujone	16.77	0.77	-
**Chrysanthenone**	17.53	**11.27**	**1.11**
Trans-pinocarveol	17.70	-	0.18
**Camphor**	18.57	**43.15**	**41.33**
Chrysanthemyl alcohol	18.89	0.33	-
**Pinocarvone**	19.04	**1.13**	0.97
**Borneol**	19.16	**1.97**	0.93
4-terpineol	19.53	0.81	0.92
Alpha-terpineol	19.97	0.23	-
Myrtenal / Myrtenol	20.21	0.59	0.78
Bornyl acetate	23.38	0.47	-
Beta -caryophyllene	26.69	0.54	0.38
Gamma-curcumene	27.64	0.37	-
Germacrene D	27.77	0.33	-

Table S3. Mean (using n = 3) content (%) of constituents in essential oil obtained by steam distillation *A. tridentata* Nutt. var. *wyomingensis* (Beetle & Young) Welsh accessions biomass collected from Bighorn Mountains in Wyoming.

Constituent	RT (mins)	Accession #223	Range
2-acetylfuran	8.71	3.23	0.48-3.23
**Santolina triene**	8.96	**6.02**	**4.53-6.02**
Tricyclene	9.50	3.39	0.33-3.39
**Alpha-pinene**	9.97	**4.04**	**1.06-4.04**
**Camphene**	10.63	**7.98**	**6.64-7.98**
5,5-dimethylfuran-2-one	10.77	3.93	0.50-3.93
**Arthole**	11.82	**17.36**	**17.36-20.15**
Yomogi alcohol	12.62	4.50	0.44-4.50
Para-cymene	13.59	4.98	0.68-4.98
**Eucalyptol**	13.97	**12.99**	**12.41-12.99**
**Cis-arbusculone**	14.67	**5.58**	**1.01-5.58**
Gamma-terpinene	14.93	5.43	0.67-5.43
**Trans-arbusculone**	15.51	**8.55**	**5.06-8.55**
**Chrysanthenone**	17.23	**6.42**	**1.01-6.42**
**Alpha-santoline alcohol**	18.34	**14.81**	**13.01-14.81**
**Camphor**	18.43	**20.55**	**20.55-21.67**
**Chrysanthemyl alcohol**	18.89	**7.05**	**1.13-7.05**
**Borneol**	19.16	**7.19**	**1.20-7.19**

Table S4. Mean (using n = 3) content (%) of constituents in essential oil obtained by steam distillation of *A. cana* Pursh var. *cana* accessions biomass collected from Bighorn Mountains in Wyoming.

Constituent	RT (mins)	Accessions #219	#220
**Santolina triene**	8.96	**3.54**	**3.69**
Tricyclene	9.50	0.41	0.39
**Alpha-pinene**	10.01	**2.72**	**3.18**
**Camphene**	10.64	**7.37**	**6.55**
**Arthole**	11.76	**9.03**	**6.24**
Dehydro-1,8-cineole	12.23	0.17	0.21
Yomogi alcohol	12.75	-	0.18
Alpha-terpinene	13.27	0.33	0.48
Para-cymene	13.61	0.63	0.72
Limonene	13.66	0.27	0.85
**Eucalyptol**	14.11	**15.26**	**20.53**
Santonia epoxide	14.73	0.67	0.62
**Gamma-terpinene**	14.97	**1.77**	**1.66**
Cis-sabinene hydrate	15.26	0.35	0.40
Artemisia alcohol	15.90	0.20	0.20
Terpinolene	16.08	-	0.22
Trans-sabinene hydrate	16.57	0.19	0.24
Chrysanthenone	17.28	0.37	-
Alpha-campholenal	17.59	0.31	0.50
**Alpha-santoline alcohol**	18.34	**11.33**	**4.78**

**Camphor**	18.48	**35.25**	**40.59**
Chrysanthemyl alcohol	18.95	0.76	0.60
Pinocarvone	19.00	0.64	0.60
**Borneol**	19.18	**2.34**	**2.64**
**4-terpineol**	19.58	**1.47**	**2.13**
Alpha-terpineol	20.02	0.44	0.86
Myrtenal / Myrtenol	20.21	0.55	0.45
Bornyl acetate	23.39	0.45	0.45

Table S5. Mean (using n = 3) content (%) of constituents in essential oil obtained by steam distillation of *A. longifolia* Nutt. accessions biomass collected from Bighorn Mountains in Wyoming.

Constituent	RT (mins)	Accessions		
		#216	#217	#218
**Santolina triene**	8.96	**2.66**	**6.79**	**3.10**
Tricyclene	9.50	0.34	0.36	0.37
**Alpha-pinene**	10.01	**2.78**	**2.82**	**3.23**
**Camphene**	10.64	**5.82**	**7.01**	**6.86**
**Arthole**	11.76	**9.7**	**6.76**	**3.22**
Dehydro-1,8-cineole	12.23	0.24	0.36	0.33
Yomogi alcohol	12.75	0.25	0.19	-
Alpha-terpinene	13.27	0.58	0.32	0.47
Para-cymene	13.61	0.35	0.92	0.52
**Limonene**	13.66	0.51	**1.33**	0.80
**Eucalyptol**	14.11	**30.79**	**24.57**	**25.48**
**Santonia epoxide**	14.73	0.65	**1.22**	0.38
**Gamma-terpinene**	14.97	**1.54**	**2.55**	**1.49**
Cis-sabinene hydrate	15.26	0.18	0.28	0.60
Artemisia alcohol	15.90	0.22	0.17	0.14
Terpinolene	16.08	0.25	-	0.22
Trans-sabinene hydrate	16.57	0.22	0.33	-
Chrysanthenone	17.28	0.42	0.25	-
Alpha-campholenal	17.59	0.46	0.43	0.37
**Alpha-santoline alcohol**	18.34	**7.60**	**6.05**	-
**Camphor**	18.48	**24.18**	**27.67**	**43.36**
**Chrysanthemyl alcohol**	18.95	**1.03**	0.40	0.26
Pinocarvone	19.00	0.43	0.33	0.89
**Borneol**	19.18	**2.29**	**2.42**	**2.44**
**4-terpineol**	19.58	**2.55**	**1.72**	**2.17**
Alpha-terpineol	20.02	0.96	0.80	0.75
Myrtenal / Myrtenol	20.21	0.52	0.46	0.51
Bornyl acetate	23.39	0.42	0.64	0.39
Germacrene D	27.76	-	0.33	-
Davana ether isomer	28.12	-	0.33	-

Table S6. Mean (using n = 3) content (%) of constituents in essential oil obtained by steam distillation of *A. ludoviciana* Nutt. *ssp. ludoviciana* accession biomass collected from Bighorn Mountains in Wyoming.

Constituent	RT (mins)	Accession
		#213
**Santolina triene**	8.96	**2.16**
Tricyclene	9.51	0.68
**Alpha-pinene**	10.07	**1.89**
**Camphene**	10.72	**13.3**
Sabinene	11.56	0.57
**Beta-pinene**	11.71	**1.43**
1-octen-3-ol	11.83	0.15
Dehydro-1,8-cineole	12.21	0.20
Alpha-terpinene	13.26	0.42
Para-cymene	13.68	0.71
**Eucalyptol**	14.04	**17.93**
Gamma-terpinene	14.94	0.81
**Artemisia ketone**	15.02	**1.27**
Cis-sabinene hydrate	15.28	0.80
Atemisia alcohol	15.88	0.23
Terpinolene	16.07	0.19
Trans-sabinene hydrate	16.51	0.47
Linalool	16.63	0.19
Chrysanthenone / Aplha-campholenal	17.54	0.28
**Camphor**	18.60	**46.18**
Pinocarvone	19.02	0.45
Cis-chrysanthemol	19.10	0.71
**Borneol**	19.24	**4.55**
**4-terpineol**	19.57	**1.29**
Alpha-terpineol	19.99	0.44
Myrtenal	20.21	0.50
Verbenone	20.66	0.21
Cis-carveol	21.36	0.23
Bornyl acetate	23.38	0.29
Artedouglasia oxide C	28.37	0.38
Artedouglasia oxide A	28.55	0.49
Artedouglasia oxide D	29.22	0.25
Artedouglasia oxide B	30.08	0.19

Table S7. Mean (using n = 3) content (%) of constituents in essential oil obtained by hydrodistillation of *A. tridentata* var. *vaseyana* accessions biomass (leaves) collected from Bighorn Mountains in Wyoming.

Constituent (in essential oil obtained from leaves)	RT (mins)	Accessions		
		#259	#261	#262
**Santolina triene**	6.67	**6.08**	**2.18**	-
Tricyclene	7.10	-	0.46	-
Alpha-pinene	7.53	0.42	2.27	-
**Camphene**	8.06	**3.30**	**12.27**	-
**Beta-pinene**	9.08	-	**1.07**	-
**Atemiseole**	9.13	-	**13.82**	-
**Artemiseole**	9.26	**42.75**	**-**	-
Yomogi alcohol	10.02	0.57	0.18	0.35
Ortho-cymene / Para-cymene	10.85	-	0.54	-
**Eucalyptol**	11.11	-	**6.77**	-
**4,8-dimethyl-trans-nona-1,3,7-triene**	11.15	**1.23**	-	-
Santolina slcohol	11.17	0.26	0.36	-
Santolina epoxide	11.81	0.11	-	-
**Unknown 1**	13.96	**4.12**	-	-
**Chrysanthenone**	14.14	-	**1.52**	-
**Alpa-santoline alcohol**	14.70	**3.34**	-	-
**Camphor**	14.79	**15.54**	-	-
**Unknown 2**	14.89	**3.23**	-	-
**Camphor**	14.95	-	**53.62**	-
Borneol	15.33	-	0.93	-
**Trans-chrysanthemol**	15.34	-	-	**3.27**
**Pinocarvone**	15.73	-	**1.46**	-
**Unknown 3**	15.78	**2.37**	-	-
**4-terpineol**	15.82	-	**1.57**	-
Myrtenol / Myrtenal	16.33	-	0.78	-
**Fragranol**	17.16	-	-	**25.5**
**Unknown 4**	17.82	**1.50**	-	-
**Unknown 5**	18.07	**1.45**	-	-
**Unknown 6**	18.17	**2.20**	-	-
Bornyl acetate	18.89	-	0.60	-
**Trans-alpha-necrodol acetate**	18.89	-	-	**9.99**
**Cyclooctadiene**	20.47	-	-	**38.32**
**Unknown 7**	21.61	-	-	**9.22**
**Unknown 8**	21.82	-	-	**3.67**
**Intermedeol**	25.32	**2.73**	-	-
**Unknown 9**	27.88	-	-	**4.96**
**Unknown 10**	27.89	**5.05**	-	-
**Unknown 11**	28.32	-	-	**3.64**
**Unknown 12**	28.33	**4.74**	-	-
Chrysanthenone/Alpha-campholenal	-	-	0.58	-

Table S8. Mean (using n = 3) content (%) of constituents in essential oil obtained by hydrodistillation of *A. tridentata* var. *wyomingensis* accessions biomass (leaves or inflorescences) collected from Bighorn Mountains in Wyoming.

Constituents (in essential oil obtained from leaves)	RT (mins)	Accessions	
		#251	#260
Cis-salveve	4.73	-	0.31
**Santolina triene**	6.68	**9.64**	-
Alpha-pinene	7.53	0.54	0.24
**Camphene**	8.06	**2.39**	**2.82**
5,5-dimethyl-2(5H)-furanone	8.23	0.22	-
Sabinene	8.98	-	0.35
Artemiseole / Beta-pinene	9.11	-	0.20
**Artemiseole**	9.24	**32.59**	-
Yomogi alcohol	10.06	0.71	-
Ortho-cymene / Para-cymene	10.79	-	0.33
Para-cymene	10.82	0.82	
**Eucalyptol**	10.99	**10.75**	**1.53**
**4,8-dimethyl-trans-nona-1,3,7-triene**	11.19	**2.33**	-
Santolina alcohol	11.27	0.37	-
Santolina epoxide	11.83	0.24	-
**Unknown 1 isomer A**	12.07	**1.79**	-
**Unknown 1 isomer B**	12.49	**3.40**	-
**Unknown 2 isomer A**	12.61	**1.36**	-
**Unknown 2 isomer B**	12.69	**1.24**	-
**Cis-thujone**	13.84	-	**71.00**
**Trans-thujone**	14.09	-	**4.54**
**Methyl -santolinate**	14.76	**8.64**	-
**Camphor**	14.84	**11.03**	**17.17**
Borneol	15.31	0.94	0.58
Pinocarvone	15.69	-	0.52
**4-terpineol**	15.77	**1.24**	0.77
Unknown 3	17.54	0.36	-
**Unknown 4 isomer A**	17.83	**1.65**	-
**Unknown 4 isomer B**	18.08	**1.26**	-
**Unknown 5**	18.58	**1.34**	-
**Constituents (in essential oil obtained from inflorescences)**			
**Santolina triene**	6.68	**7.29**	-
Tricyclene	7.10	0.09	-
Alpha-pinene	7.54	0.55	-
**Camphene**	8.08	**3.24**	-
5,5-dimethyl-2(5H)-furanone	8.23	0.22	-
**Artemiseole**	9.24	**26.34**	-
Yomogi alcohol	10.06	0.68	-
Para-cymene	10.82	0.74	-
**Eucalyptol**	11.08	**14.01**	-
**4,8-dimethyl-trans-nona-1,3,7-triene**	11.19	**2.47**	-
Santolina alcohol	11.27	0.28	-
Santolina epoxide	11.83	0.26	-
Gamma-terpinene	11.98	0.26	-
**Unknown 1 isomer A**	12.07	**2.92**	-
**Unknown 1 isomer B**	12.49	**5.89**	-

Unknown 2 isomer A	12.61	0.80	-
Unknown 2 isomer B	12.69	0.90	-
Chrysanthenone	14.13	0.33	-
**Methyl -santolinate**	14.76	**10.13**	-
**Camphor**	14.85	**13.70**	-
Borneol	15.31	0.78	-
**4-terpineol**	15.79	**1.59**	-
**Unknown 3**	16.52	**1.20**	-
Unknown 4	17.54	0.81	-
**Unknown 5 isomer A**	17.83	**1.27**	-
Unknown 5 isomer B	18.08	0.33	-
Unknown 6	18.58	0.93	-

Table S9. Mean (using n = 3) content (%) of constituents in essential oil obtained by hydrodistillation of *A. cana* var. *cana* accessions biomass (leaves or inflorescences) collected from Bighorn Mountains in Wyoming.

**Constituents (in essential oil obtained from leaves)**	**RT (mins)**	**Accessions**						
		**#252**	**#253**	**#254**	**#255**	**#256**	**#257**	**#258**
**Santolina triene**	6.67	**2.20**	0.48	-	**1.61**	**2.76**	**4.65**	-
**Tricyclene**	7.10	0.35	**2.95**	0.39	0.28	0.32	0.35	0.43
**Alpha-pinene**	7.55	**2.07**	-	**2.48**	**2.47**	**3.12**	**2.28**	**3.30**
**Camphene**	8.10	**7.48**	0.92	**7.66**	**6.92**	**6.74**	**5.48**	**11.34**
**Sabinene**	8.98	-	**16.23**	0.18	-	-	-	-
**Beta-pinene**	9.08	**1.32**	-	**2.00**	**1.35**	**1.83**	**1.23**	**2.07**
**Atemiseole**	9.13	**5.39**	**1.30**	-	**4.19**	**3.97**	**9.83**	-
**Yomogi alcohol**	10.07	0.32	**5.05**	-	0.23	0.56	0.42	-
**Alpha-phellandrene**	10.08	-	**17.11**	0.39	-	-	-	-
Alpha-terpinene	10.51	-	-	0.31	-	0.22	-	0.29
**Ortho-cymene / Para-cymene**	10.85	**2.16**	**31.20**	**4.85**	**2.00**	**2.77**	**2.44**	**4.04**
**Eucalyptol**	11.11	**30.47**	0.63	**20.64**	**25.37**	**22.26**	**25.45**	**24.32**
**Santolina alcohol**	11.17	0.32	**9.03**	-	-	0.20	0.66	-
**Trans-ocimene**	11.65	-	**2.48**	-	-	-	-	-
Santolina epoxide	11.82	0.42	0.18	-	0.21	0.44	0.42	-
Prenyl isobutanoate	11.92	-	0.33	-	-	-	-	-
**Gamma-terpinene**	11.98	0.50	**5.83**	0.68	0.33	0.64	0.35	0.67
Alpha-campholenal	13.96	-	0.23	-	0.30	-	-	0.65
Chrysanthenone	14.14	-	-	0.47	-	-	-	-
**Camphor**	14.95	**40.57**	-	**48.55**	**47.64**	**45.16**	**37.16**	**42.95**
**Borneol**	15.33	1.32	-	**1.59**	0.98	**1.01**	**1.19**	**1.50**
**Pinocarvone**	15.73	-	-	**2.04**	0.60	**2.09**	**2.33**	**1.67**
**4-terpineol**	15.82	3.22	-	**2.63**	**2.89**	**3.25**	**3.30**	**3.09**
Alpha-terpineol	16.22	-	-	0.79	0.76	0.99	0.92	-
Myrtenol / Myrtenal	16.33	0.76	-	0.85	-	0.66	-	0.77
**Bornyl acetate**	18.89	-	0.68	**2.18**	0.80	0.98	0.73	**2.97**
Alpha-terpinyl acetate	20.43	-	-	-	-	-	-	-
**Chrysanthenone/Alpha-campholenal**	-	0.67	0.35	**1.53**	0.60	-	0.63	-
**Constituents (in essential oil obtained from inflorescences)**								
**Santolina triene**	6.67	**2.21**	0.41	-	**1.43**	**3.10**	**9.05**	-
**Tricyclene**	7.10	0.39	**2.56**	0.49	0.38	0.38	0.30	0.61
**Alpha-pinene**	7.55	**1.71**	-	**3.03**	**3.39**	**3.04**	**2.75**	**5.37**

**Camphene**	8.10	**8.46**	0.87	**9.72**	**8.17**	**8.31**	**6.19**	**14.68**
**Sabinene**	8.98	-	**15.84**	0.18	-	0.18	-	0.24
**Beta-pinene**	9.08	**1.02**	-	**2.13**	**1.58**	**1.63**	**1.31**	**2.88**
**Atemiseole**	9.13	**7.53**	**1.11**	-	**3.11**	**4.14**	**8.48**	-
**Yomogi alcohol**	10.07	0.27	**5.25**	-	0.28	0.24	0.39	-
**Alpha-phellandrene**	10.08	-	**16.28**	0.99	-	-	-	-
Alpha-terpinene	10.51	-	-	0.26	-	-	-	0.25
**Ortho-cymene / Para-cymene**	10.85	**2.33**	**28.39**	**6.35**	**1.90**	**3.32**	**2.01**	**4.57**
**Eucalyptol**	11.11	**29.79**	-	**21.67**	**23.56**	**21.4**	**24.66**	**21.02**
**Santolina alcohol**	11.17	0.35	**8.72**	-	-	0.27	0.57	-
**Trans-ocimene**	11.65	-	**3.60**	-	-	-	-	-
Santolina epoxide	11.82	0.84	0.20	-	0.47	0.24	0.73	-
Prenyl isobutanoate	11.92	-	0.88	-	-	-	0.16	-
**Gamma-terpinene**	11.98	**1.00**	**10.90**	0.77	0.53	0.52	0.88	0.89
Alpha-campholenal	13.96	-	0.25	-	-	-	-	-
**Chrysanthenone**	14.14	-	**1.20**	0.42	0.26	0.36	-	-
**Camphor**	14.95	**38.7**	-	**45.36**	**47.76**	**43.68**	**36.13**	**40.49**
**Borneol**	15.33	1.18	-	**1.20**	0.89	0.94	0.74	**1.09**
**Pinocarvone**	15.73	0.86	-	**1.19**	-	**1.35**	**1.58**	**1.57**
**4-terpineol**	15.82	2.58	-	**2.13**	**3.06**	**2.84**	**2.99**	**2.54**
Alpha-terpineol	16.22	-	-	0.67	0.85	0.91	0.86	-
Myrtenol / Myrtenal	16.33	0.77	-	0.62	0.59	0.67	-	0.58
**Bornyl acetate**	18.89	-	0.80	**1.25**	0.79	0.85	-	**3.20**
**Alpha-terpinyl acetate**	20.43	-	-	-	-	**1.16**	-	-
Chrysanthenone/Alpha-campholenal		-	0.38	0.87	0.64	0.59	-	-

Table S10. Mean (using n = 3) content (%) and range of EO obtained by hydrodistillation for accessions from Bighorn Mountains in Wyoming in the Fall of 2014.

Species	Accession	Leaves		Inflorescences	
		Mean	Range	Mean	Range
Artemisia spp.	250	0.79	0.69 - 0.90	1.08	1.00 - 1.14
A. tridentata var. *wyomingensis*	251	0.71	0.53 - 1.06	1.15	1.06 - 1.22
A. cana var. *cana*	252	0.49	0.45 - 0.52	0.56	0.43 - 0.64
A. cana var. *cana*	253	0.51	0.48 - 0.58	0.28	0.17 - 0.34
A. cana var. *cana*	254	0.08	0.04 - 0.11	0.30	0.28 - 0.34
A. cana var. *cana*	255	0.63	0.59 - 0.67	0.86	0.73 – 1.00
A. cana var. *cana*	256	0.90	0.81 - 0.96	0.96	0.84 - 1.10
A. cana var. *cana*	257	0.50	0.42 - 0.60	0.91	0.77 - 1.02
A. cana var. *cana*	258	0.15	0.13 - 0.17	0.40	0.30 - 0.53
A. tridentata var. *vaseyana*	259	0.16	0.12 - 0.18	-	-
A. tridentata var. *wyomingensis*	260	1.06	1.00 - 1.16	-	-
A. tridentata var. *vaseyana*	261	0.31	0.24 - 0.36	-	-
A. tridentata var. *vaseyana*	262	0.22	0.06 - 0.42	-	-

Table S11. Evaluation of select essential oils against *Leishmania donovani* and *Plasmodium falciparum* D6. Data is percent inhibition. Primary evaluations performed at 80 ug/mL for *L. donovani* and 15,867 ng/mL for *P. falciparum* D6.

Species	*L. donovani*	*P. falciparum* D6
A. cana var. *cana*	0	0
A. longifolia	0	34
A. tridentate var. *tridentata*	3	0
A. tridentata var. *vaseyana*	4	16
A. tridentata var. *wyomingensis*	13	14

Table S12. Evaluation of select essential oils against opportunistic infection pathogens. Primary evaluations performed at 50 ug/mL and data is percent inhibition.

Species	*Aspergillus fumigatus*	*Candida albicans*	*Candida glabrata*	*Candida krusei*	*Crypto-coccus nefor-mans*	*E. coli*	*M. intra-cellu-lare*	*MRS*	*Pseudo-monas aerugi-nosa*	*Staphylo-coccus aureus*
A. cana var. *cana*	8	0	6	1	13	2	0	3	2	4
A. longifolia	9	0	5	0	9	2	0	1	0	1
A. tridentate var. *tridentata*	11	0	6	3	18	2	0	3	4	5
A. tridentata var. *vaseyana*	4	0	3	2	4	0	4	0	3	0
A. tridentata var. *wyomingensis*	12	0	5	8	21	3	0	0	6	7
